# The feasibility of deep learning-based synthetic contrast-enhanced CT from nonenhanced CT in emergency department patients with acute abdominal pain

**DOI:** 10.1038/s41598-021-99896-4

**Published:** 2021-10-14

**Authors:** Se Woo Kim, Jung Hoon Kim, Suha Kwak, Minkyo Seo, Changhyun Ryoo, Cheong-Il Shin, Siwon Jang, Jungheum Cho, Young-Hoon Kim, Kyutae Jeon

**Affiliations:** 1 Department of Radiology, Seoul National University Hospital, 101 Daehak-ro, Jongno-gu, Seoul, 03080 Republic of Korea; 2 Department of Radiology, Seoul National University College of Medicine, 101 Daehangno, Jongno-gu, Seoul, 03080 Republic of Korea; 3 Department of Computer Science and Engineering, POSTECH, 77 Cheongam-Ro, Nam-Gu, Pohang-si, Gyeongbuk, 37673 Republic of Korea; 4 Department of Radiology, Boramae Medical Center, 20 Boramae-ro 5-gil, Dongjak-gu, Seoul, 07061 Republic of Korea; 5 Department of Radiology, Seoul National University Bundang Hospital, 82 Gumi-ro 173 Beon-gil, Bundang-gu, Seongnam-si, Gyeonggi-do 13620 Republic of Korea

**Keywords:** Medical research, Digestive signs and symptoms

## Abstract

Our objective was to investigate the feasibility of deep learning-based synthetic contrast-enhanced CT (DL-SCE-CT) from nonenhanced CT (NECT) in patients who visited the emergency department (ED) with acute abdominal pain (AAP). We trained an algorithm generating DL-SCE-CT using NECT with paired precontrast/postcontrast images. For clinical application, 353 patients from three institutions who visited the ED with AAP were included. Six reviewers (experienced radiologists, ER1-3; training radiologists, TR1-3) made diagnostic and disposition decisions using NECT alone and then with NECT and DL-SCE-CT together. The radiologists’ confidence in decisions was graded using a 5-point scale. The diagnostic accuracy using DL-SCE-CT improved in three radiologists (50%, *P* = 0.023, 0.012, < 0.001, especially in 2/3 of TRs). The confidence of diagnosis and disposition improved significantly in five radiologists (83.3%, *P* < 0.001). Particularly, in subgroups with underlying malignancy and miscellaneous medical conditions (MMCs) and in CT-negative cases, more radiologists reported increased confidence in diagnosis (83.3% [5/6], 100.0% [6/6], and 83.3% [5/6], respectively) and disposition (66.7% [4/6], 83.3% [5/6] and 100% [6/6], respectively). In conclusion, DL-SCE-CT enhances the accuracy and confidence of diagnosis and disposition regarding patients with AAP in the ED, especially for less experienced radiologists, in CT-negative cases, and in certain disease subgroups with underlying malignancy and MMCs.

## Introduction

Since its introduction in the 1970s, the use of computed tomography (CT) has increased in various clinical settings, including emergency departments (EDs)^1^. In particular, CT-associated ED visits have increased dramatically in patients older than 65 years and in patients with acute abdominal pain (AAP)^1^. According to a previous study, in patients who visited the ED complaining of AAP, CT may make substantial contributions to diagnosis or disposition decisions and may confirm or exclude alternative diagnoses^2^. Because physician diagnostic accuracy and confidence increase with CT^2^, CT plays a critical role in the diagnosis and management of patients with AAP in the ED^1–3^.

Intravenous (IV) contrast agents are widely used in CT examination and are known to improve sensitivity and specificity in many indications^4^. However, the risk of adverse events, including allergies and nephropathy, must be considered before administering IV contrast agents^5,6^. In patients with advanced age and underlying chronic kidney disease, the risk of contrast-associated acute kidney injury is increased^7,8^. A nationwide survey revealed that the proportion of noncontrast-enhanced CT (NECT) among all abdominal CTs has increased from 9 to 14%, and among all abdominal NECTs, the proportion of examinations in patients over 65 years of age has increased from 31 to 41% over the past 10 years^9^. Since not all patients who visit the ED complaining of AAP are indicated for contrast-enhanced CT (CECT), improving the diagnostic performance of NECT is important.

Recently, deep learning algorithms that reduce the dose of contrast agent or synthesize virtual contrast-enhancement images have been technically validated in brain MRI^10,11^. One researcher proposed the technical feasibility of synthesizing virtual contrast-enhancement of heart chambers from NECT using a deep learning algorithm^12^. However, to the best of our knowledge, no previous study has validated the clinical utility of virtual contrast-enhanced abdominal CT synthesized by a deep learning algorithm. The purpose of our study was to investigate the clinical feasibility of deep learning-based synthetic contrast-enhanced CT (DL-SCE-CT) from NECT in patients who visited the ED complaining of AAP.

## Results

### Baseline demographics and clinical characteristics of the test dataset

The mean age of the included patients was 57.3 years (187 male and 166 female patients). The number of patients in each subgroup was as follows: acute pancreatitis (N = 20), acute diverticulitis (N = 21), liver disease (N = 26), biliary disease (N = 23), oncologic condition (N = 42), acute appendicitis (N = 21), bowel obstruction (N = 22), miscellaneous surgical condition (MSC) (N = 35), miscellaneous medical condition (MMC) (N = 59), and nonspecific abdominal pain (NSAP) (N = 84). Miscellaneous surgical conditions included bowel perforation, bowel strangulation, acute mesenteric ischemia, common hepatic artery pseudoaneurysm after pancreatectomy, acute aortic syndrome and ovarian cyst rupture. Miscellaneous medical conditions included urinary tract infection, urolithiasis, enterocolitis, past or active gastrointestinal bleeding, peptic ulcer and intraabdominal abscess requiring percutaneous drainage. NSAP included cases without a demonstrable cause of abdominal pain on CT.

### Outcomes of clinical validation of deep learning-based synthetic CT Images

#### Step 1: Review of diagnostic performance of image analysis with NECT alone

With NECT alone, the accuracy of diagnosis ranged from 69.4 to 81.5%, and the accuracy of the disposition decision ranged from 70.1 to 84.4%. The accuracy of both diagnosis and disposition decisions differed according to the dataset and reviewer expertise. The accuracy of diagnosis was superior for the experienced radiologists (76.5–81.5%) compared to that of the training radiologists (69.4–77.1%) and in selectively enrolled datasets with specific diagnoses (Dataset-A, 75.5–87.0%) compared to that in consecutively enrolled datasets (Dataset-B, 61.4–73.2%). Similarly, the accuracy of the disposition decision was better in ERs (76.8–84.4%) than in TRs (70.3–75.1%) and in Dataset-A (76.0–91.5%) than in Dataset-B (60.1–75.2%). The confidence of diagnosis and dispositions was equivocal regardless of reviewer expertise (ERs: 3.47–4.07 and 3.83–4.08; TRs: 2.87–4.09 and 3.92–4.19) or dataset (Dataset-A: 3.12–4.18 and 3.97–4.26, Dataset-B: 2.55–4.03 and 3.64–4.11, respectively).

#### Step 2: Review of diagnostic performance of image analysis with the aid of DL-SCE-CT

**Figure 1:**
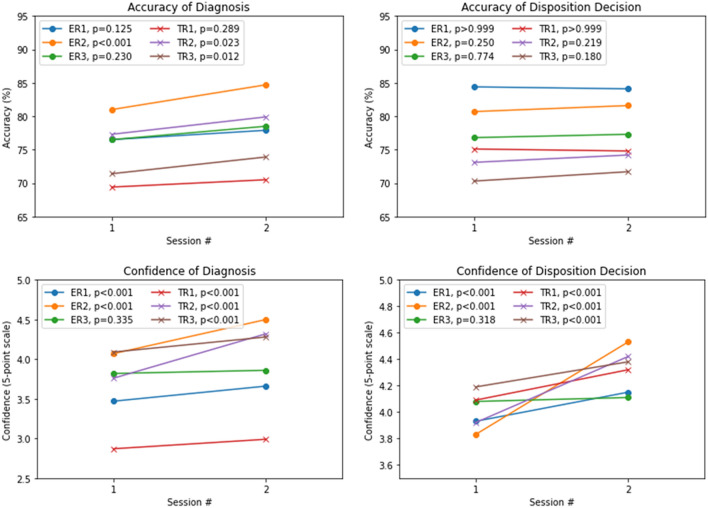
The accuracies and confidences of diagnosis and disposition decisions in each radiologist in 1st and 2nd sessions of image review. The accuracies of diagnosis show increasing tendency in 2nd session (statistically significant increase observed in three of the radiologists and two of the training radiologists). The accuracies of disposition decision show equivocal change between two sessions. The confidences of diagnosis and disposition decision both shows statistically significant increases in five of the six radiologists. ER, experienced radiologist; TR, training radiologist.

**Figure 2:**
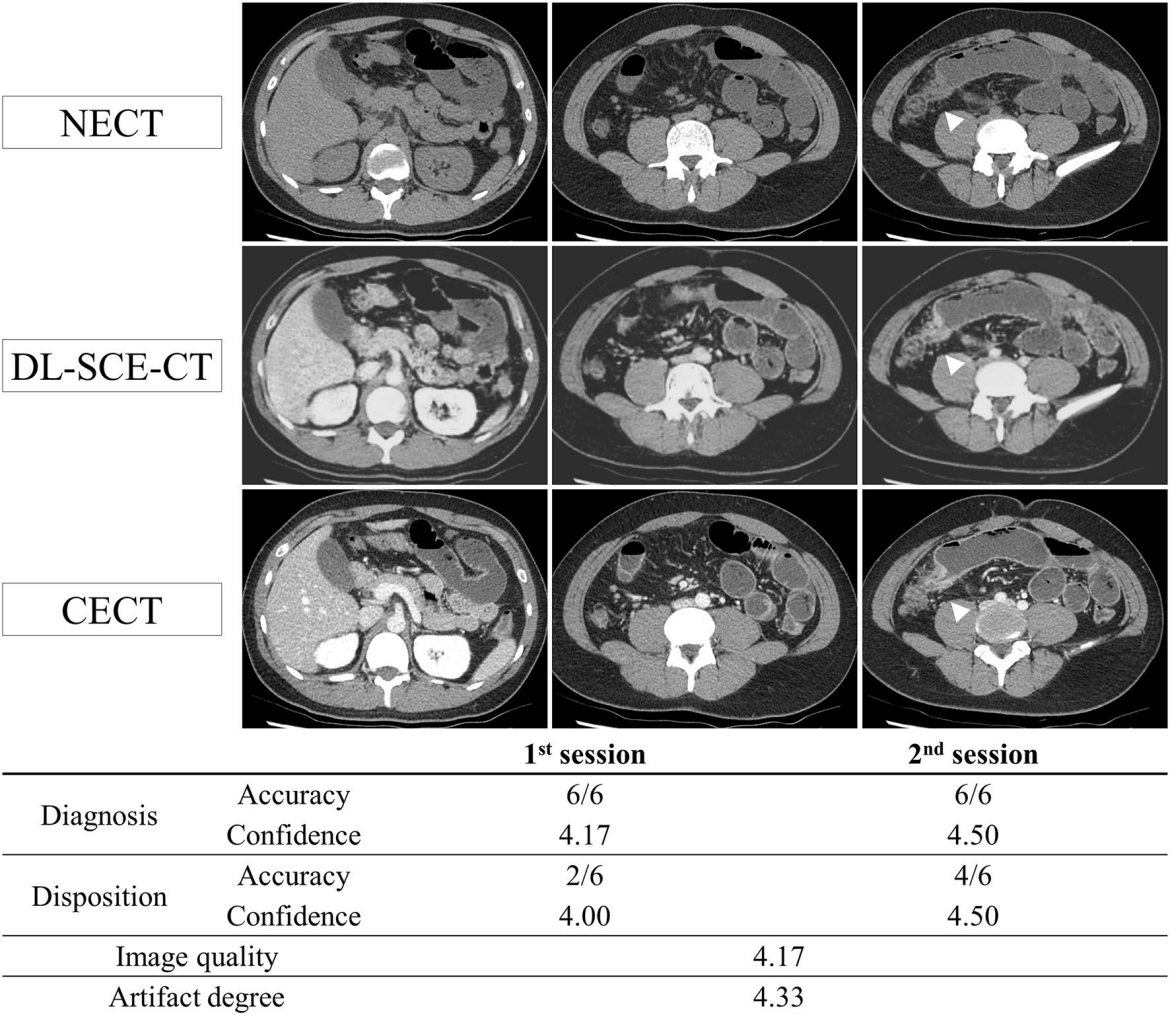
A 25-year-old male patient who visited the ED complaining of abdominal pain. CT images show fluid distension of small bowel loops with transition at the terminal ileum (arrowhead). The contrast among the bowel wall, visceral fat, and intraluminal fluid is more evident in DL-SCE-CT than in NECT. The patient was admitted for management of Crohn’s disease flares. In this case, all of reviewers made the correct diagnosis (small bowel obstruction at terminal ileum) regardless of DL-SCE-CT. However, two more radiologists made correct disposition decision (admission for medical management) after review of DL-SCE-CT. Moreover, with the aid of DL-SCE-CT, the confidence of the diagnosis and disposition decision increased from 4.17 to 4.50 and 4.00 to 4.50, respectively.

**Figure 3:**
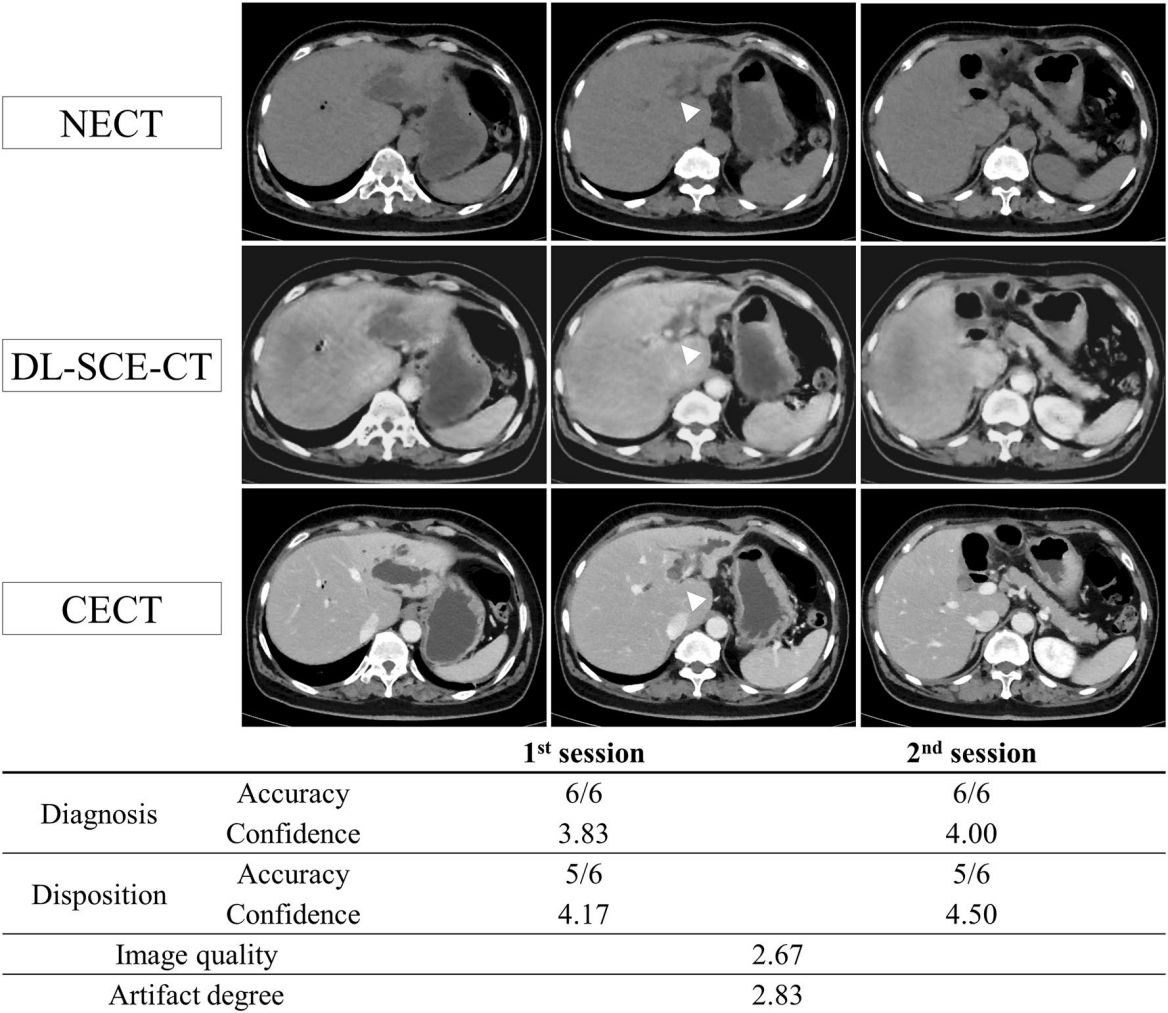
A 65-year-old female patient who visited the ED complaining of abdominal pain and fever. CT images show intrahepatic duct stones (arrowhead) with dilated upstream bile ducts. The contrast among the liver parenchyma, fluid within the dilated bile duct, and stones within the bile duct are more evident in DL-SCE-CT than in NECT. The patient was admitted for management of obstructive cholangiohepatitis. In this case, 100.0% (6/6) and 83.3% (5/6) of radiologists made the correct diagnoses and disposition decisions (intrahepatic duct stones with biliary obstruction, admission for medical management), regardless of DL-SCE-CT. However, both radiologists’ confidence in the diagnosis and disposition decisions improved from 3.83 to 4.00 and 4.17 to 4.50, respectively, with the aid of DL-SCE-CT.

Table 1 and Fig. 1 summarize the diagnostic performance of radiologists with or without the aid of DL-SCE-CT. Overall, with the aid of DL-SCE-CT, the accuracy of diagnosis increased from 69.4–81.0% to 70.5–84.7%. Of the six radiologists, three radiologists (50%, *P* = 0.023, 0.012, < 0.001) reported a significant increase in diagnostic accuracy with DL-SCE-CT. In particular, two-thirds of TRs experienced significant improvement in accuracy. The confidence of diagnosis (from 2.87–4.09 to 2.99–4.50) and disposition (from 3.83–4.19 to 4.11–4.53) also increased, with statistically significant increments observed by five of the six radiologists (83.3%, *P* < 0.001) (Figs. 2, 3). The accuracy of the disposition decision did not show a significant change for any radiologist. The diagnostic performance of each radiologist with or without the aid of DL-SCE-CT is shown in Supplementary Tables S1 to S6.

**Table 1:** Accuracy and Confidence of Diagnosis and Disposition decisions.

		Accuracy of diagnosis		Confidence of diagnosis		Accuracy of disposition		Confidence of disposition	
		1st session	2nd session	1st session	2nd session	1st session	2nd session	1st session	2nd session
Total	Range^†^	69.4–81.0	70.5–84.7	2.87–4.09	2.99–4.50	70.3–84.4	71.7–84.1	3.83–4.19	4.11–4.53
	P values^‡^	0.125, < ***0.001***, 0.230, 0.289, ***0.023***, ***0.012***		< ***0.001***, < ***0.001***, 0.335, < ***0.001***, < ***0.001***, < ***0.001***		> 0.999, 0.250, 0.774, > 0.999, 0.219, 0.180		< ***0.001***, < ***0.001***, 0.318, < ***0.001***, < ***0.001***, < ***0.001***	
Dataset-A	Range^†^	75.5–87.0	77.0–92.5	3.12–4.18	3.20–4.62	76.0–91.5	77.0–91.5	3.97–4.26	4.21–4.60
	P values^‡^	0.063, ***0.001***, 0.424, 0.25, 0.180, ***0.040***		0.375, < ***0.001***, 0.528, ***0.01***, < ***0.001***, < ***0.001***		> 0.999, 0.500, > 0.999, > 0.999, 0.500, 0.453		< ***0.001***, < ***0.001***, 0.867, < ***0.001***, < ***0.001***, < ***0.001***	
Dataset-B	Range^†^	61.4–73.2	62.1–75.8	2.55–4.03	2.71–4.36	60.1–75.2	61.4–74.5	3.64–4.11	3.90–4.44
	P values^‡^	> 0.999, 0.500, 0.549, > 0.999, 0.125, 0.500		< ***0.001***, < ***0.001***, ***0.041***, < ***0.001***, < ***0.001***, < ***0.001***		> 0.999, > 0.999, 0.727, > 0.999, 0.625, 0.500		< ***0.001***, < ***0.001***, 0.073, < ***0.001***, < ***0.001***, < ***0.001***	

^†^The range of accuracies (%) and arithmetic means of confidence (5-point scale) reported by six radiologists.

^‡^Numbers are *P* values reported by each radiologist. McNemar’s test and Wilcoxon test were performed for each radiologist between 1st and 2nd sessions for comparison of accuracy and confidence, respectively.

*Bold italics* indicate statistical significance.

### Subgroup analysis according to disease category

The accuracy and confidence of diagnosis and disposition decisions were variably reported in different subgroups and are summarized in Table 2. Disease categories in which more than half of the radiologists experienced an increase in confidence of both diagnosis and disposition decisions included oncologic conditions (5/6: 83.3% and 4/6: 66.7%, respectively), MMCs (6/6: 100.0% and 5/6: 83.3%, respectively), and NSAP (5/6: 83.3% and 6/6: 100.0%, respectively). For acute pancreatitis, acute diverticulitis, biliary disease, and acute appendicitis, more than half of the radiologists showed no significant change in confidence in diagnosis and disposition decisions. The confidence of diagnosis and disposition decisions in the 1st session were generally lower in useful subgroups (oncologic conditions, MMC, and NSAP, 1.31–4.71 and 2.81–4.77, respectively) than in less useful subgroups (acute pancreatitis, acute diverticulitis, biliary disease, and acute appendicitis, 3.48–4.90 and 3.70–4.90, respectively). There was no particular subgroup in which DL-SCE-CT could significantly improve more than half of the radiologists’ accuracy of diagnosis or disposition.

**Table 2:** Subgroup Analysis by Disease Category.

	Accuracy of diagnosis		Confidence of diagnosis		Accuracy of disposition		Confidence of disposition	
	1st session	2nd session	1st session	2nd session	1st session	2nd session	1st session	2nd session
Acute pancreatitis (N = 20)	70.0–100.0	70.0–100.0	4.35–4.75	4.40–4.75	95.0–100.0	95.0–100.0	4.35–4.90	4.52–4.90
	(NA, NA, > 0.999, > 0.999, NA, NA)		(NA, ***0.028***, NA, NA, ***0.018***, NA)		(NA, NA, > 0.999, > 0.999, NA, NA)		(NA, ***0.012***, NA, NA, ***0.028***, NA)	
Acute diverticulitis (N = 21)	81.0–100.0	81.0–100.0	4.29–4.91	4.52–4.90	81.0–95.2	76.2–95.2	4.43–4.95	4.52–4.90
	(0.500, 0.125, 0.375, > 0.999, 0.219, 0.625)		(NA, NA, NA, NA, ***0.028***, NA)		(NA, NA, NA, > 0.999, NA, > 0.999)		(NA, ***0.012***, NA, NA, ***0.028***, NA)	
Liver disease (N = 26)	26.9–53.8	23.1–69.2	2.00–3.54	1.92–4.31	50.0–76.9	46.2–76.9	3.23–3.69	3.23–4.38
	(0.500, 0.125, 0.375, > 0.999, 0.219, 0.625)		(0.813, < ***0.001***, 0.013*, 0.625, ***0.001***, ***0.012***)		(> 0.999, NA, > 0.999, > 0.999, 0.500, > 0.999)		(> 0.999, < ***0.001***, 0.161, 0.078, ***0.006***, ***0.043***)	
Biliary disease (N = 23)	65.2–95.7	65.2–95.7	3.48–4.30	3.65–4.43	39.1–91.3	47.8–91.3	3.70–4.30	4.09–4.44
	(NA, 0.500, 0.500, NA, NA, > 0.999)		(NA, ***0.001***, 0.735, 0.125, ***0.010***, NA)		(NA, > 0.999, > 0.999, NA, NA, 0.500)		(NA, < ***0.001***, 0.345, NA, ***0.022***, NA)	
Oncologic condition (N = 42)	45.2–71.4	47.6–85.7	2.83–3.98	3.12–4.64	71.4–81.0	71.4–81.0	3.50–4.55	3.71–4.71
	(NA, ***0.031***, 0.070, > 0.999, 0.625, 0.500)		(***0.027***, < ***0.001***, 0.709, ***0.003***, ***0.001***, ***0.001***)		(> 0.999, > 0.999, NA, > 0.999, > 0.999, > 0.999)		(> 0.999, < ***0.001***, 0.401, ***0.031***, < ***0.001***, ***0.006***)	
Acute appendicitis (N = 21)	85.7–100.0	85.7–100.0	4.05–4.81	4.48–4.81	85.7–100.0	85.7–100.0	4.00–4.81	4.52–4.85
	(NA, NA, NA, NA, NA, > 0.999)		(NA, ***0.012***, NA, NA, ***0.008***, NA)		(NA, NA, NA, NA, NA, NA)		(NA, ***0.005***, 0.361, NA, ***0.008***, NA)	
Bowel obstruction (N = 22)	81.8–95.5	86.4–95.5	3.68–4.77	3.91–4.77	54.5–90.9	54.5–90.9	3.64–4.77	4.09–4.82
	(NA, NA, NA, NA, NA, > 0.999)		(NA, ***0.012***, 0.068, NA, ***0.002***, NA)		(> 0.999, NA, > 0.999, NA, NA, NA)		(> 0.999, < ***0.001***, 0.068, NA, ***0.005***, ***0.043***)	
MSC ^†^ (N = 35)	60.0–77.1	62.9–80.0	3.51–4.71	4.00–4.77	51.4–91.4	54.3–88.6	3.69–4.80	4.03–4.86
	(NA, > 0.999, > 0.999, NA 0.500, > 0.999)		(NA, ***0.043***, 0.610, ***0.002***, ***0.003***, ***0.006***)		(NA, NA, > 0.999, > 0.999, 0.500, > 0.999)		(NA, < ***0.001***, NA, 0.063, ***0.013***, ***0.013***)	
MMC^‡^ (N = 59)	61.0–81.4	57.6–81.4	2.88–4.36	3.00–4.54	45.8–81.4	42.4–78.0	3.71–4.41	3.97–4.58
	(> 0.999, NA, 0.688, > 0.999, > 0.999, NA)		(**< *0.001***, < ***0.001***, ***0.049***, ***0.0391***, < ***0.001***, ***0.005***)		(0.500, > 0.999, > 0.999, 0.500, NA, NA)		(0.5, < ***0.001***, 0.702, < ***0.001***, < ***0.001***, ***0.008***)	
NSAP (N = 84)	72.6–96.4	76.2–96.4	1.31–4.37	1.30–4.50	75.0–95.2	78.6–95.2	2.81–4.43	3.43–4.63
	(0.250, NA, NA, 0.500, NA, 0.500)		(< ***0.001***, < ***0.001***,** 0.001**, 0.875, < ***0.001***, ***0.029***)		(0.375, NA, NA, 0.500, NA, 0.500)		(0.375, < ***0.001***, < ***0.001***, < ***0.001***, < ***0.001***, ***0.014***)	

NOTE. NA = not available, MSC = miscellaneous surgical condition, MMC = miscellaneous medical condition, NSAP = nonspecific abdominal pain.

Numbers are ranges of accuracy or confidence of diagnosis and disposition reported by six radiologists in 1st and 2nd sessions of image review. Numbers in parentheses are *P* values. McNemar’s test and Wilcoxon test were performed for each radiologist between 1st and 2nd sessions for comparison of accuracy and confidence, respectively.

^†^Miscellaneous surgical condition includes bowel perforation, bowel strangulation, acute mesenteric ischemia, common hepatic artery pseudoaneurysm after pancreas resection, acute aortic syndrome and ovarian cyst rupture.

^‡^Miscellaneous medical condition includes urinary tract infection, urinary tract stone, enterocolitis, past or active upper or lower GI bleeding, peptic ulcer and intraabdominal abscess requiring percutaneous drainage.

*One of experienced radiologists (ER3) reported statistically significant decrease of diagnostic confidence in 2nd session compared to 1st session (3.15 in the 1st session and 2.73 in the 2nd session, *P* value = 0.013).

The radiologists determined that the image quality of DL-SCE-CT was sufficient (with moderate limitations for clinical use but no substantial loss of information; mean score 3.33), and the artifact degree was moderate (with preserved diagnostic reliability; mean score 3.25). The image quality score ranged from 1.83 to 4.17, and the artifact degree score ranged from 2.00 to 4.33.

## Discussion

In our study, DL-SCE-CT was feasible and helpful for patients visiting the ED with complaints of AAP, increasing the radiologists’ accuracy of diagnosis and confidence level in diagnosis and disposition decisions. In particular, DL-SCE-CT was useful in cases with oncologic conditions, MMCs, and NSAP and for less experienced radiologists. The confidence of diagnosis and disposition decisions significantly increased in five of the radiologists (83.3%, *P* < 0.001). In addition, diagnostic accuracy was significantly improved in half of the radiologists (*P* = 0.023, 0.012, < 0.001). In particular, DL-SCE-CT was more helpful in training radiologists, improving the diagnostic accuracy of two-thirds of the radiologists (*P* = 0.023 and 0.012). Technically, the image quality of DL-SCE-CT was rated as sufficient with moderate limitations and without substantial loss of information, and the degree of artifact was rated as moderate with preserved diagnostic reliability.

DL-SCE-CT was more helpful in oncologic conditions, MMCs and NSAP (helpful subgroups) but less useful in the acute pancreatitis, acute diverticulitis, biliary disease, and acute appendicitis subgroups (unhelpful subgroups). In diseases belonging to the unhelpful subgroups, diagnosis often depends on findings such as fat strandings in the organ-fat interface (e.g., peripancreatic, periappendiceal, or peridiverticular fat strandings) or radio-opaque stones (e.g., acute calculous cholecystitis or cholangitis), which are easily detected by NECT. In these less useful subgroups, radiologists showed high confidence in diagnosis and disposition decisions using NECT alone (3.48–4.91 and 3.23–4.95). In contrast, radiologists showed lower confidence for NECT evaluation of helpful subgroups (1.31–4.36 and 2.81–4.55). It is meaningful that DL-SCE-CT increases the confidence of radiologists in diseases that are difficult to diagnose using NECT alone.

Recently, deep-learning-based synthetic medical images have been an active area of research with broad applications in various medical disciplines. Attempts to increase patient safety using deep learning-based contrast dose reduction have been made. Some researchers have accomplished reductions in the gadolinium dose used for brain MRI using a deep learning method^10^, while other researchers synthesized fake contrast enhancement images for brain MRI and cardiac CT^11,12^. Technically, unlike other previous algorithms, our algorithm was able to minimize the misalignment issue caused by minute breathing and patient movement between the input data NECT image and the reference standard CECT image using a two-stage approach^27^. A detailed description of our algorithm is provided in the Methods section.

Moreover, consideration of clinical significance is necessary for such technology to be used in real-world situations. AAP is one of the most common reasons for visiting the ED, accounting for up to 7–10% of all ED visits^13,14^. Diseases causing AAP range from self-limiting to life-threatening conditions^14^, causing large medical and socioeconomic burdens^15,16^. In particular, the overall burden of AAP and difficulty of diagnosis increase with advancing age^14^^;^^16–18^. Thus, the number and proportion of CT-associated ED visits has rapidly increased in elderly patients with AAP ^1–3^^;^^19–21^. However, liberal use of CT is accompanied by an increased risk of ionizing radiation exposure^22,23^ and adverse effects due to IV contrast agents^5,6,8^. Thus, the results of our study are valuable in this situation by augmenting the diagnostic performance of NECT and radiologists’ confidence in decision making, although more improvements are warranted in the future.

Our study has several limitations. First, there is inherent selection bias owing to the retrospective nature of our study. A considerable portion (56.7%, 200/353) of our study population was selectively enrolled, increasing selection bias. Second, the improvement was greater in confidence than in the accuracy of radiologists’ decisions. Although increased confidence is a meaningful benefit, DL-SCE-CT should improve radiologists’ actual performance to improve the clinical outcomes in patients. We hope to improve clinical outcomes by further elaborating the quality of synthetic contrast enhancement. Third, the increments of confidence were present in both correct and incorrect cases, raising concern for increasing confidence of misdiagnosis or mistreatment.

Deep learning-based synthetic CT (DLSCT) might be developed and applied in various clinical settings. Synthetic contrast enhancement is only one of many possibilities. Various kinds of image augmentation could potentially improve patient outcomes. Appropriate clinical settings are necessary for the development of useful synthetic images. For elderly patients with decreased renal function, both an NECT-based synthetic enhancement method and a method that uses a small dose of contrast agent but mimics the use of a full dose (e.g. as used in brain MRI^10^) could be developed. A combination with pre-existing contrast dose reduction technologies, such as dual energy CT, might be attempted. For particularly radiosensitive populations, such as pediatric patients, deep-learning-based denoising algorithms might be especially helpful^24–26^ to facilitate ultralow-dose imaging. Therefore, further investigation of potentially useful DLSCTs is warranted.

### Conclusion

In conclusion, according to our preliminary study, DL-SCE-CT is feasible and is helpful for more accurate and confident diagnosis and disposition decisions regarding patients with AAP in the ED. In particular, DL-SCE-CT is useful in cases with oncologic conditions, MMCs, negative CT findings, and for less experienced radiologists.

## Methods

This retrospective multicenter study was approved by the joint Institutional Review Board of Seoul National University Hospital, Seoul National University Bundang Hospital, and Boramae Medical Center. The Institutional Review Board granted a waiver of informed patient consent due to the retrospective nature of our study. All methods were performed in accordance with the relevant guidelines and regulations.

### Study population and image dataset

**Figure 4:**
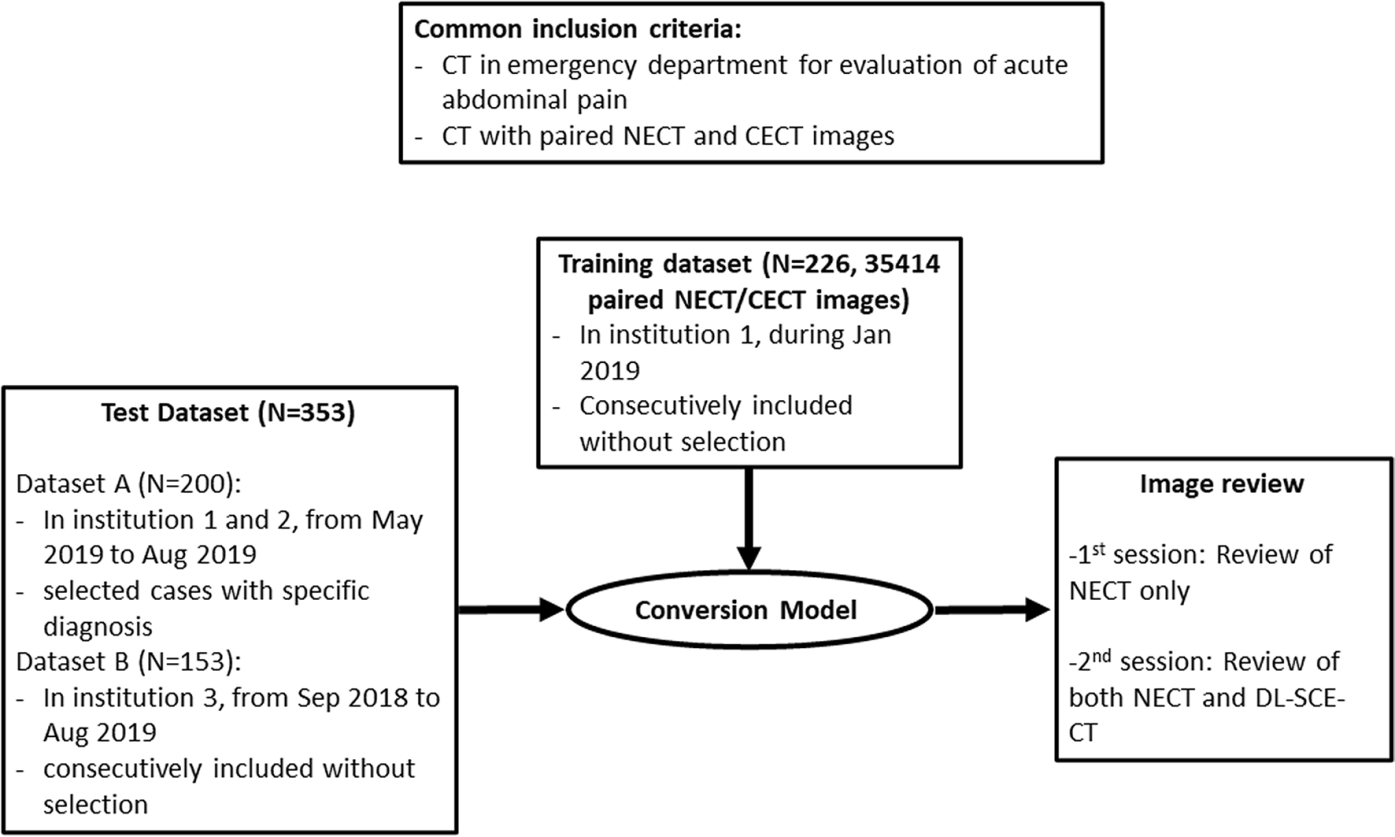
Flow diagram of the study design and study population inclusion process. NECT, nonenhanced CT; CECT, contrast-enhanced CT; DL-SCE-CT, deep learning-based synthetic contrast-enhanced CT.

We trained the conversion model using a pre-existing algorithm that generates DL-SCE-CT from NECT^27^. We used a training dataset consisting of 226 consecutive CT examinations (35,414 paired NECT and CECT images) that were performed in the ED of a tertiary hospital for the evaluation of AAP in January 2019. Then, two test datasets were prepared for the clinical validation of DL-SCE-CT. Common inclusion criteria for both datasets were as follows: (1) CT examinations performed in the ED for the evaluation of AAP and (2) CT examinations consisting of paired NECT and CECT images. Then, among the CT examinations performed in two institutions (one tertiary and one secondary hospital) from May 2019 to August 2019, one radiologist (S.W.K. with 5 years of experience in abdominal radiology) selected 200 CT exams, either with one of the following specific diagnoses (N = 159): biliary disease, acute appendicitis, acute diverticulitis, acute pancreatitis, oncologic pain, miscellaneous surgical condition, bowel obstruction and liver disease, or nonspecific findings (N = 41) as Dataset-A^13^. Among the CT examinations performed in another institution (tertiary hospital) from January 2019 to June 2019, 153 CT cases meeting the common inclusion criteria were consecutively included as Dataset-B. The NECT and subsequently generated DL-SCE-CT using the aforementioned conversion model were included in both datasets. Figure 4 summarizes the inclusion process of the study population.

For each case, one radiologist (S.W.K.) meticulously reviewed the CT images and electronic medical records, including the clinicopathologic data and laboratory test results, to determine the most appropriate diagnosis and disposition decisions (admission for medical treatment, admission for surgical treatment, or discharge).

### CT techniques

All CT examinations were conducted using MDCT scanners with 64–160 detector rows. The acquisition parameters were as follows: tube voltage, 100–120 kVp; tube current, 50–300 mAs; slice thickness, 2.0–3.0 mm; reconstruction interval, 1.0–3.0 mm; pitch, 0.5–1.3; and rotation time, 0.33–0.75 s. Iodinated contrast agent (320 or 350 mg I/mL) was injected using an automatic power injector at a rate of 2.0–5.0 mL/s (total amount 1.6–2.0 mL/kg). The portal phase acquisition time was determined by either the bolus tracking method (beginning of portal phase scan 45–60 s after reaching threshold attenuation [100 HU] at the descending thoracic aorta or immediately after reaching threshold attenuation [100 HU] at the hepatic parenchyma) or fixed time delay (90 s after contrast injection).

### Development of DL-SCE-CT image

**Figure 5:**
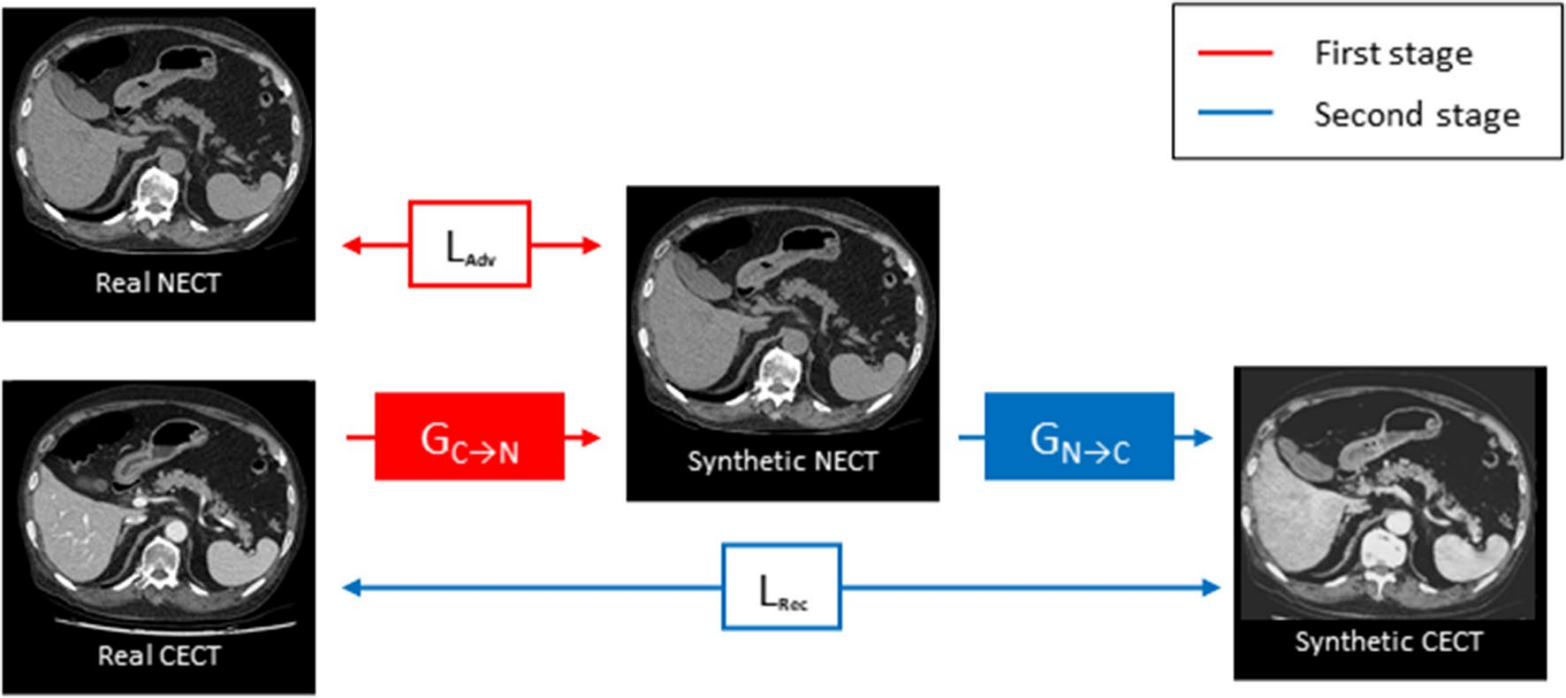
Schematic diagram of the two-stage approach used for making the conversion model. In the first stage, the generator (G_C→N_), which generates synthetic NECT from real CECT, is trained adversarially using a conditional generative adversarial network. In the second stage, another generator (G_N→C_) that generates synthetic CECT from NECT is trained using a deep convolutional neural network. During the second stage of training, synthetic NECT, which is generated from and perfectly aligned with real CECT, is used as input data, resolving the misregistration issue between input data and ground truth (real CECT). NECT, nonenhanced CT; CECT, contrast-enhanced CT; L_Adv_, adversarial loss; L_rec_, reconstruction loss; G_C→N_, generator that generates synthetic NECT from real CECT; G_N→C_, generator that generates synthetic CECT from NECT.

A conversion model that generates DL-SCE-CT from NECT was developed in a previous study using 23,923 paired NECT and CECT images from 327 CT examinations^27^. In our study, we adopted the same model with identical architecture and trained it using our training dataset (226 CT examinations with 35,414 paired NECT and CECT images). In contrast to other neural image syntheses, a major problem in synthetic contrast enhancement of abdominal CT is inevitable misalignment between NECT and CECT images owing to patients’ breathing and involuntary movements during examinations. Our model was developed using a two-stage approach to overcome the misalignment issue using a conditional generative adversarial network (cGAN) and a deep convolutional neural network (CNN). In the first stage, a generator (G_C→N_) that creates synthetic NECT from real CECT was trained adversarially with a discriminator (D) that distinguishes among synthetic NECT, real NECT and real CECT. This stage, which is an inverse of our target task, is technically much easier because NECT images are much less patient-specific than CECT images due to monotonic intensities. The resulting synthetic NECT, which is perfectly aligned with real CECT, is used in the second stage for training a generator (G_N→C_), which creates synthetic CECT from synthetic NECT. Generators (G_C→N_ and G_N→C_) were trained using the SPADE architecture, one of the state-of-the-art methods in image-to-image translation. The second generator (G_N→C_) was finally used to create our synthetic images in the test dataset (Datasets A and B). Figure 5 shows the development process.

### Clinical validation of DL-SCE-CT

#### Step 1: Image analysis using NECT alone

Six reviewers with different expertise participated in two image review sessions (ER1: C.I.S.; ER2: J.H.K.; and ER3: S.J., board-certified experienced abdominal radiologists with 16, 23, and 7 years of experience, respectively; TR1: C.H.R.; TR2: J.C.; TR3: K.J.; training radiologists with 4, 5, and 4 years of experience, respectively). In the first session, the reviewers were asked to report the diagnosis in subjective form and the disposition decision in three-option multiple choice format (admission for medical treatment, admission for surgical treatment, and discharge) based on NECT alone. Reviewers were aware that the images being reviewed were from patients who visited the ED with AAP, but no further clinical or laboratory data were provided. The reviewers’ confidence in the diagnosis and disposition decision was rated on a 5-point scale (1: Not confident at all; 2: Slightly confident; 3: Somewhat confident; 4: Fairly confident; and 5: Completely confident).

#### Step 2: Image analysis with the aid of DL-SCE-CT

The second review session was initiated two weeks after the first session ended. In the second session, the reviewers were asked to report the diagnosis and disposition decision after reviewing both NECT and DL-SCE-CT with the 5-point scale confidence level. In addition, the reviewers were asked to report the image quality (1: poor image quality-image not usable; 2: restricted image quality-severe limitations for clinical use; clear loss of information; 3: sufficient image quality-moderate limitations for clinical use but no substantial loss of information; 4: good image quality-minimal limitations for clinical use; and 5: excellent image quality-no limitations for clinical use) and artifact degree (1: artifacts resulting in a nondiagnostic image; 2: severe artifacts resulting in limited diagnostic reliability; 3: moderate degree with preserved diagnostic reliability; 4: minimal degree with preserved diagnostic reliability; and 5: excellent without artifact) of DL-SCE-CT on a 5-point scale. The arithmetic means of scores rated by six radiologists were used as representative image quality and artifact degree scores for each case.

### Statistical analysis

The accuracy of the diagnosis and disposition decisions were compared between the two review sessions using McNemar’s test. Confidence in the diagnosis and disposition decisions were compared using the Wilcoxon test. For subgroup analysis, the whole study population was divided into ten subgroups according to the CT diagnosis: acute pancreatitis, acute diverticulitis, liver disease (e.g., acute hepatitis and liver abscess), biliary disease (e.g., acute cholecystitis and acute cholangitis), oncologic condition (e.g., malignant bowel obstruction, malignant biliary obstruction and ruptured HCC with hemoperitoneum), acute appendicitis, bowel obstruction, miscellaneous surgical conditions, miscellaneous medical conditions, and NSAP. The accuracy and confidence were separately evaluated in each subgroup. A commercially available software package (MedCalc Statistical Software, Version 19.2.1, MedCalc Software) was used for statistical analysis. A *p value* less than 0.05 was considered statistically significant.

## Supplementary Information

Supplementary Information.

## Data Availability

The datasets generated and/or analyzed during the current study are available from the corresponding author upon reasonable request. However, our Institutional Review Board prohibits open the data relate to patient’s personal medical information and images.
