# Supplementary material for: The feasibility of deep learning-based synthetic contrast-enhanced CT from nonenhanced CT in emergency department patients with acute abdominal pain
Source: Sci Rep. 2021 Oct 14;11:20390. doi: 10.1038/s41598-021-99896-4 (PMC8516935; doi:10.1038/s41598-021-99896-4)
Supplement: Supplementary file 1 — Supplementary Information. [file 41598_2021_99896_MOESM1_ESM.pdf]

The feasibility of deep learning-based synthetic contrast-enhanced CT from nonenhanced CT in emergency department patients with acute abdominal pain

Se Woo Kim[1], Jung Hoon Kim[1,2,*], Suha Kwak[3], Minkyo Seo[3], Changhyun Ryoo[1], Cheong Il Shin[1,2], Siwon Jang[4], Jungheum Cho[5], Young Hoon Kim[2,5], Kyutae Jeon[1]

Department of Radiology, Seoul National University Hospital[1] and Seoul National University College of Medicine[2], 101 Daehangno, Jongno-gu, Seoul, 03080, Korea

Department of Computer Science and Engineering, POSTECH[3], 77 Cheongam-Ro, Nam-Gu, Pohang-si, Gyeongbuk, 37673, Korea

Department of Radiology, Boramae Medical Center[4], 20 Boramae-ro 5-gil, Dongjak-gu, Seoul, 07061, Korea

Department of Radiology, Seoul National University Bundang Hospital[5], 82 Gumi-ro 173 Beon-gil, Bundang-gu, Seongnam-si, Gyeonggi-do, 13620, Korea

Corresponding Author:

Jung Hoon Kim, MD, PhD

Department of Radiology

Seoul National University College of Medicine, Seoul National University Hospital

101 Daehak-ro, Jongno-gu, Seoul 03080, Republic of Korea

Tel: 82-2-2072-1969

Fax: 82-2-743-6385

E-mail: jhkim2008@gmail.com

Supplemental Material

Supplementary table S1. Summary table of image review by ER1

	Diagnostic Accuracy†			Diagnostic Confidence‡			Disposition accuracy†			Disposition Confidence‡		
	1st session	2nd session	P-value	1st session	2nd session	P-value	1st session	2nd session	P-value	1st session	2nd session	P-value
Total (N=353)	76.5	77.9	0.125	3.47	3.66	***<0.001***	84.4	84.1	>0.999	3.93	4.15	***<0.001***
Dataset-A (N=200)	84.0	86.5	0.063	3.70	3.68	0.375	91.5	91.5	>0.999	4.15	4.34	***<0.001***
Dataset-B (N=153)	66.0	66.7	>0.999	3.17	3.64	***<0.001***	75.2	74.5	>0.999	3.64	3.90	***<0.001***
AP (N=20)	95.0	95.0	NA	4.75	4.75	NA	100.0	100.0	NA	4.70	4.80	NA
AD (N=21)	100.0	100.0	NA	4.90	4.90	NA	95.2	95.2	NA	4.95	4.90	NA
LD (N=26)	38.5	46.2	0.500	2.73	2.65	0.813	76.9	73.1	>0.999	3.23	3.38	0.376
BD (N=23)	95.7	95.7	NA	4.30	4.26	NA	91.3	91.3	NA	4.30	4.17	NA
OC (N=42)	52.4	52.4	NA	3.29	3.50	***0.027***	81.0	81.0	>0.999	3.64	3.71	0.461
AA (N=21)	100.0	100.0	NA	4.71	4.81	NA	100.0	100.0	NA	4.76	4.85	NA
BO (N=22)	95.5	95.5	NA	4.73	4.68	NA	90.9	86.4	>0.999	4.68	4.82	NA
MSC (N=35)	71.4	71.4	NA	4.71	4.77	NA	88.6	88.6	NA	4.80	4.86	NA
MMC (N=59)	81.4	81.4	>0.999	3.80	4.17	***<0.001***	81.4	78.0	0.5	4.17	4.34	***0.002***
NSAP (N=84)	72.6	76.2	0.250	1.51	1.95	***<0.001***	75.0	78.6	0.375	2.81	3.46	***<0.001***

NOTE. **ER** = experienced radiologist, **AP** = acute pancreatitis, **AD** = acute diverticulitis, **LD** = liver disease, **BD** = biliary disease, **OC** = oncologic condition, **AA** = acute appendicitis, **BO** = bowel obstruction, **MSC** = miscellaneous surgical condition, **MMC** = miscellaneous medical condition, **NSAP** = nonspecific abdominal pain, **NA** = not available

†The numbers are percentages of radiologist's accuracy of diagnosis or disposition decision during 1st and 2nd session of image review. McNemar's test was performed for comparison between two sessions.

‡ The numbers are arithmetic means of radiologist's confidence level (rated as 5-point-scale in each case) of diagnosis or disposition decision during 1st and 2nd session of image review. Wilcoxon test was performed for comparison between two sessions.

Bold italics indicate statistical significance

Supplementary table S2. Summary table of image review by ER2

	Diagnostic Accuracy†			Diagnostic Confidence‡			Disposition accuracy†			Diagnostic Confidence‡		
	1st session	2nd session	P-value	1st session	2nd session	P-value	1st session	2nd session	P-value	1st session	2nd session	P-value
Total (N=353)	81.0	84.7	***<0.001***	4.07	4.50	***<0.001***	80.7	81.6	0.250	3.83	4.53	***<0.001***
Dataset-A (N=200)	87.0	92.5	***0.001***	4.10	4.62	***<0.001***	89.0	90.0	0.500	3.97	4.60	***<0.001***
Dataset-B (N=153)	73.2	74.5	0.500	4.03	4.36	***<0.001***	69.9	70.6	>0.999	3.65	4.44	***<0.001***
AP (N=20)	100.0	100.0	NA	4.40	4.70	***0.028***	100.0	100.0	NA	4.35	4.75	***0.012***
AD (N=21)	100.0	100.0	NA	4.67	4.81	NA	90.5	90.5	NA	4.43	4.81	***0.012***
LD (N=26)	53.8	69.2	0.125	3.54	4.31	***<0.001***	76.9	76.9	NA	3.46	4.38	***<0.001***
BD (N=23)	82.6	91.3	0.500	3.78	4.39	***0.001***	73.9	78.3	>0.999	3.70	4.43	***<0.001***
OC (N=42)	71.4	85.7	***0.031***	3.98	4.64	***<0.001***	78.6	81.0	>0.999	3.79	4.71	***<0.001***
AA (N=21)	95.2	95.2	NA	4.05	4.48	***0.012***	95.2	95.2	NA	4.00	4.52	***0.005***
BO (N=22)	90.9	90.9	NA	4.36	4.77	***0.012***	77.3	77.3	NA	3.64	4.36	***<0.001***
MSC (N=35)	74.3	77.1	>0.999	4.60	4.74	***0.043***	71.4	71.4	NA	4.23	4.74	***<0.001***
MMC (N=59)	61.0	61.0	NA	4.10	4.41	***<0.001***	59.3	61.0	>0.999	3.71	4.44	***<0.001***
NSAP (N=84)	95.2	95.2	NA	3.80	4.31	***<0.001***	94.0	94.0	NA	3.65	4.39	***<0.001***

NOTE. **ER** = experienced radiologist, **AP** = acute pancreatitis, **AD** = acute diverticulitis, **LD** = liver disease, **BD** = biliary disease, **OC** = oncologic condition, **AA** = acute appendicitis, **BO** = bowel obstruction, **MSC** = miscellaneous surgical condition, **MMC** = miscellaneous medical condition, **NSAP** = nonspecific abdominal pain, **NA** = not available

†The numbers are percentages of radiologist's accuracy of diagnosis or disposition decision during 1st and 2nd session of image review. McNemar's test was performed for comparison between two sessions.

‡ The numbers are arithmetic means of radiologist's confidence level (rated as 5-point-scale in each case) of diagnosis or disposition decision during 1st and 2nd session of image review. Wilcoxon test was performed for comparison between two sessions.

Bold italics indicate statistical significance

Supplementary table S3. Summary table of image review by ER3

	Diagnostic Accuracy†			Diagnostic Confidence‡			Disposition accuracy†			Diagnostic Confidence‡		
	1st session	2nd session	P-value	1st session	2nd session	P-value	1st session	2nd session	P-value	1st session	2nd session	P-value
Total (N=353)	76.5	78.5	0.230	3.82	3.86	0.335	76.8	77.3	0.774	4.08	4.11	0.318
Dataset-A (N=200)	82.5	84.5	0.424	4.06	4.03	0.528	85.5	85.5	>0.999	4.22	4.21	0.867
Dataset-B (N=153)	68.6	70.6	0.549	3.52	3.65	***0.041***	65.4	66.7	0.727	3.90	3.99	0.073
AP (N=20)	95.0	100.0	>0.999	4.70	4.65	NA	95.0	100.0	>0.999	4.90	4.80	NA
AD (N=21)	95.2	95.2	NA	4.91	4.86	NA	90.5	90.5	NA	4.91	4.86	NA
LD (N=26)	34.6	46.2	0.375	3.15	2.73	***0.013***	50.0	46.2	>0.999	3.46	3.23	0.161
BD (N=23)	78.3	69.6	0.500	4.04	4.00	0.735	82.6	78.3	>0.999	4.22	4.09	0.345
OC (N=42)	66.7	81.0	0.070	3.79	3.74	0.709	71.4	71.4	NA	4.55	4.48	0.401
AA (N=21)	95.2	95.2	NA	4.81	4.67	NA	95.2	95.2	NA	4.81	4.71	0.361
BO (N=22)	95.5	95.5	NA	4.77	4.50	0.068	77.3	81,8	>0.999	4.77	4.55	0.068
MSC (N=35)	77.1	80.0	>0.999	4.23	4.29	0.610	80.0	82.9	>0.999	4.74	4.77	NA
MMC (N=59)	61.0	57.6	0.688	3.61	3.81	***0.049***	61.0	62.7	>0.999	3.93	3.97	0.702
NSAP (N=84)	85.7	85.7	NA	3.00	3.30	***0.001***	83.3	83.3	NA	3.06	3.43	***<0.001***

NOTE. **ER** = experienced radiologist, **AP** = acute pancreatitis, **AD** = acute diverticulitis, **LD** = liver disease, **BD** = biliary disease, **OC** = oncologic condition, **AA** = acute appendicitis, **BO** = bowel obstruction, **MSC** = miscellaneous surgical condition, **MMC** = miscellaneous medical condition, **NSAP** = nonspecific abdominal pain, **NA** = not available

†The numbers are percentages of radiologist's accuracy of diagnosis or disposition decision during 1st and 2nd session of image review. McNemar's test was performed for comparison between two sessions.

‡ The numbers are arithmetic means of radiologist's confidence level (rated as 5-point-scale in each case) of diagnosis or disposition decision during 1st and 2nd session of image review. Wilcoxon test was performed for comparison between two sessions.

Bold italics indicate statistical significance

Supplementary table S4. Summary table of image review by TR1

	Diagnostic Accuracy†			Diagnostic Confidence‡			Disposition accuracy†			Diagnostic Confidence‡		
	1st session	2nd session	P-value	1st session	2nd session	P-value	1st session	2nd session	P-value	1st session	2nd session	P-value
Total (N=353)	69.4	70.5	0.289	2.87	2.99	***<0.001***	75.1	74.8	>0.999	4.09	4.32	***<0.001***
Dataset-A (N=200)	75.5	77.0	0.250	3.12	3.20	***0.010***	85.0	85.0	>0.999	4.20	4.34	***<0.001***
Dataset-B (N=153)	61.4	62.1	>0.999	2.55	2.71	***<0.001***	62.1	61.4	>0.999	3.93	4.29	***<0.001***
AP (N=20)	90.0	95.0	>0.999	4.35	4.40	NA	100.0	95.0	>0.999	4.60	4.65	NA
AD (N=21)	81.0	81.0	NA	4.29	4.52	NA	85.7	90.5	>0.999	4.52	4.52	NA
LD (N=26)	26.9	23.1	>0.999	2.00	1.92	0.625	50.0	46.2	>0.999	3.65	3.88	0.078
BD (N=23)	65.2	65.2	NA	3.48	3.65	0.125	69.6	69.6	NA	4.17	4.17	NA
OC (N=42)	45.2	47.6	>0.999	2.83	3.12	***0.003***	73.8	73.8	>0.999	3.90	4.05	***0.031***
AA (N=21)	95.2	95.2	NA	4.38	4.48	NA	95.2	95.2	NA	4.67	4.71	NA
BO (N=22)	86.4	86.4	NA	3.77	3.91	NA	90.9	90.9	NA	4.05	4.09	NA
MSC (N=35)	74.3	74.3	NA	3.71	4.00	***0.002***	91.4	88.6	>0.999	4.62	4.77	0.063
MMC (N=59)	61.0	62.7	>0.999	2.88	3.00	***0.039***	45.8	42.4	0.5	3.83	4.14	***<0.001***
NSAP (N=84)	81.0	83.3	0.5	1.31	1.30	0.875	81.0	83.3	0.5	3.87	4.39	***<0.001***

NOTE. **TR** = training radiologist, **AP** = acute pancreatitis, **AD =** acute diverticulitis, **LD =** liver disease, **BD =** biliary disease, **OC =** oncologic condition, **AA =** acute appendicitis, **BO =** bowel obstruction, **MSC =** miscellaneous surgical condition, **MMC** = miscellaneous medical condition, **NSAP =** nonspecific abdominal pain, **NA =** not available

†The numbers are percentages of radiologist's accuracy of diagnosis or disposition decision during 1st and 2nd session of image review. McNemar's test was performed for comparison between two sessions.

‡ The numbers are arithmetic means of radiologist's confidence level (rated as 5-point-scale in each case) of diagnosis or disposition decision during 1st and 2nd session of image review. Wilcoxon test was performed for comparison between two sessions.

Bold italics indicate statistical significance

Supplementary table S5. Summary table of image review by TR2

	Diagnostic Accuracy†			Diagnostic Confidence‡			Disposition accuracy†			Diagnostic Confidence‡		
	1st session	2nd session	P-value	1st session	2nd session	P-value	1st session	2nd session	P-value	1st session	2nd session	P-value
Total (N=353)	77.3	79.9	0.023	3.76	4.32	***<0.001***	73.1	74.2	0.219	3.92	4.42	***<0.001***
Dataset-A (N=200)	80.5	83.0	0.180	3.87	4.42	***<0.001***	76.0	77.0	0.500	4.10	4.57	***<0.001***
Dataset-B (N=153)	73.2	75.8	0.125	3.60	4.19	***<0.001***	69.3	70.6	0.625	3.68	4.24	***<0.001***
AP (N=20)	90.0	90.0	NA	4.35	4.70	***0.018***	95.0	95.0	NA	4.65	4.95	***0.028***
AD (N=21)	85.7	85.7	NA	4.43	4.71	***0.028***	81.0	81.0	NA	4.57	4.86	***0.028***
LD (N=26)	30.8	46.2	0.219	3.12	3.85	***0.001***	53.8	61.5	0.500	3.69	4.19	***0.006***
BD (N=23)	78.3	78.3	NA	3.96	4.43	***0.010***	65.2	65.2	NA	3.96	4.35	***0.022***
OC (N=42)	69.0	73.8	0.625	3.36	3.79	***0.001***	73.8	73.8	0.999	3.71	4.12	***<0.001***
AA (N=21)	85.7	85.7	NA	4.33	4.76	***0.008***	85.7	85.7	NA	4.38	4.81	***0.008***
BO (N=22)	90.9	90.9	NA	3.68	4.32	***0.002***	54.5	54.5	NA	3.68	4.18	***0.005***
MSC (N=35)	71.4	77.1	0.500	3.51	4.06	***0.003***	51.4	57.1	0.500	3.71	4.03	***0.013***
MMC (N=59)	64.4	66.1	>0.999	3.80	4.34	***<0.001***	57.6	57.6	NA	3.85	4.34	***<0.001***
NSAP (N=84)	96.4	96.4	NA	3.76	4.50	***<0.001***	95.2	95.2	NA	3.82	4.63	***<0.001***

NOTE. **TR** = training radiologist, **AP** = acute pancreatitis, **AD =** acute diverticulitis, **LD =** liver disease, **BD =** biliary disease, **OC =** oncologic condition, **AA =** acute appendicitis, **BO =** bowel obstruction, **MSC =** miscellaneous surgical condition, **MMC** = miscellaneous medical condition, **NSAP =** nonspecific abdominal pain, **NA =** not available

†The numbers are percentages of radiologist's accuracy of diagnosis or disposition decision during 1st and 2nd session of image review. McNemar's test was performed for comparison between two sessions.

‡ The numbers are arithmetic means of radiologist's confidence level (rated as 5-point-scale in each case) of diagnosis or disposition decision during 1st and 2nd session of image review. Wilcoxon test was performed for comparison between two sessions.

Bold italics indicate statistical significance

Supplementary table S6. Summary table of image review by TR3

	Diagnostic Accuracy†			Diagnostic Confidence‡			Disposition accuracy†			Diagnostic Confidence‡		
	1st session	2nd session	P-value	1st session	2nd session	P-value	1st session	2nd session	P-value	1st session	2nd session	P-value
Total (N=353)	71.4	73.9	***0.012***	4.09	4.28	***<0.001***	70.3	71.7	0.180	4.19	4.38	***<0.001***
Dataset-A (N=200)	75.5	79.0	***0.040***	4.18	4.30	***<0.001***	78.0	79.6	0.453	4.26	4.40	***<0.001***
Dataset-B (N=153)	66.0	67.3	0.500	3.98	4.26	***<0.001***	60.1	61.4	0.500	4.11	4.35	***<0.001***
AP (N=20)	70.0	70.0	NA	4.60	4.70	NA	95.0	95.0	NA	4.70	4.75	NA
AD (N=21)	85.7	85.7	NA	4.57	4.62	NA	81.0	76.2	>0.999	4.76	4.86	NA
LD (N=26)	34.6	42.3	0.625	3.12	3.46	***0.012***	57.7	57.7	>0.999	3.39	3.62	***0.043***
BD (N=23)	73.9	78.3	>0.999	4.30	4.35	NA	39.1	47.8	0.500	4.30	4.44	NA
OC (N=42)	47.6	52.4	0.500	3.12	3.50	***0.001***	76.2	78.6	>0.999	3.50	3.79	***0.006***
AA (N=21)	95.2	95.2	>0.999	4.67	4.76	NA	90.5	90.5	NA	4.71	4.81	NA
BO (N=22)	81.8	86.4	>0.999	4.41	4.50	NA	72.7	72.7	NA	4.18	4.41	***0.043***
MSC (N=35)	60.0	62.9	>0.999	3.60	4.00	***0.006***	51.4	54.3	>0.999	3.69	4.03	***0.013***
MMC (N=59)	66.1	66.1	NA	4.36	4.54	***0.005***	52.5	52.5	NA	4.41	4.58	***0.008***
NSAP (N=84)	90.5	92.9	0.500	4.37	4.48	***0.029***	85.7	88.1	0.500	4.43	4.57	***0.014***

NOTE. **TR** = training radiologist, **AP** = acute pancreatitis, **AD =** acute diverticulitis, **LD =** liver disease, **BD =** biliary disease, **OC =** oncologic condition, **AA =** acute appendicitis, **BO =** bowel obstruction, **MSC =** miscellaneous surgical condition, **MMC** = miscellaneous medical condition, **NSAP =** nonspecific abdominal pain, **NA =** not available

†The numbers are percentages of radiologist's accuracy of diagnosis or disposition decision during 1st and 2nd session of image review. McNemar's test was performed for comparison between two sessions.

‡ The numbers are arithmetic means of radiologist's confidence level (rated as 5-point-scale in each case) of diagnosis or disposition decision during 1st and 2nd session of image review. Wilcoxon test was performed for comparison between two sessions.

Bold italics indicate statistical significance
